# 

**Published:** 1905-09

**Authors:** 


### CONTENTS.

ORIGINAL COMMUNICATIONS—

RECURRENT PARALYSIS OF OCULAR MUSCLES ASSOCIATED WITH PAIN. By Alex. W. Stirling, M.D., C.M. (Edin.), D. P. H. (Lond.) ..... 361
TUBERCULOSIS. By J. W. Hurt, M.D., Atlanta, Ga ..... 368

SOCIETY REPORTS—

THE SURGICAL TREATMENT OF CANCER ..... 373

EDITORIAL NOTES AND COMMENTS—

THE YELLOW FEVER SITUATION IN NEW ORLEANS ..... 377
OPENING OF WESLEY MEMORIAL HOSPITAL ..... 379
DEATH OF DR. R. W. HYNDS ..... 380

NEWS AND NOTES ..... 381
CONTENTS CONTINUED ..... X

